# A pulse check for trends in sea turtle numbers across the globe

**DOI:** 10.1016/j.isci.2024.109071

**Published:** 2024-02-15

**Authors:** Graeme C. Hays, Gail Schofield, Maria Papazekou, Anastasia Chatzimentor, Stelios Katsanevakis, Antonios D. Mazaris

**Affiliations:** 1 School of Life and Environmental Sciences, Deakin University, Geelong, VIC 3280, Australia; 2 School of Biological and Behavioural Sciences, Queen Mary University of London, London E1 4NS, UK; 3 Department of Ecology, School of Biology, Aristotle University of Thessaloniki, 54124 Thessaloniki, Greece; 4 Department of Marine Sciences, University of the Aegean, 81100 Mytilene, Greece

**Keywords:** Nature conservation, Ecology, Zoology

## Abstract

Population declines of vertebrates are common, but rebuilding marine life may be possible. We assessed trends in sea turtle numbers globally, building 61 time series of abundance extending beyond 2015, representing monitoring in >1200 years. Increases were widespread with significant upward trends, no significant change, and significant downward trends in 28, 28, and 5 time series, respectively. For example, annual nest numbers increased between 1980 and 2018 from around 4,000 to 16,000 for green turtles at Aldabra (Seychelles, Indian Ocean) and between 2008 and 2020 from around 500 to 35,000 for loggerhead turtles in Sal (Cape Verde, north Atlantic). However, conservation concerns remain. Major populations may experience declines, such as loggerhead turtles in Oman, while previous upward trends can be reversed, as with green turtles nesting at Tortuguero (Costa Rica, Caribbean). Further, decreases in abundance were evident in several leatherback turtle time series. These concerns show there is no room for complacency for sea turtle conservation.

## Highlights

• Trends in sea turtle numbers globally compiled from >1200 years of monitoring
• Increases or stable numbers were widespread and dominated
• Conservation concerns remain, with some notable decline in abundance
• Concerns show there is no room for complacency for sea turtle conservation

## Graphical abstract

**Figure:**
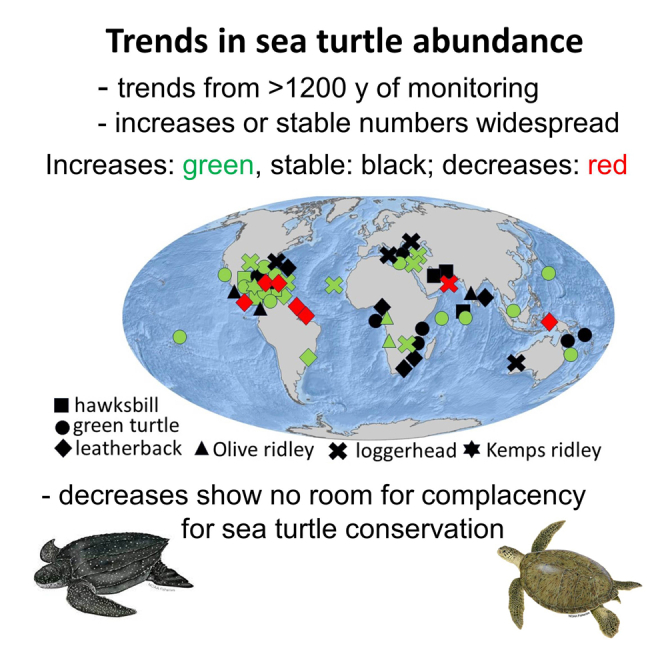

## Introduction

There are well-known threats to the world’s biodiversity that have driven many species toward extinction. Threats pervade across both marine and terrestrial systems, such as habitat loss, pollution, climate change, and harvesting^1^ and are contributing to what has been widely described as the Earth’s sixth mass extinction.^2^ However, set against the general backdrop of declines in species abundance, there are some notable exceptions of species recoveries that have suggested that marine life might be rebuilt.^3^ For example, there are many shining lights of marine mammal recovery including humpback whales migrating from Antarctica to Australia increasing from a few 100 to over 40,000 in the last 50 years, northern elephant seals increasing from around 20 individuals in 1880 to >200,000 today, and sea otters on the Pacific coasts of the USA and Canada increasing from around 50 individuals in 1911 to many 1000s currently.^3^ Sea turtles are another notable conservation success story, with levels of abundance at many nesting sites, and across multiple species, rising rather than falling, likely as a consequence of conservation measures, such as reduced harvesting of eggs and adults.^4^ However, these conservation successes should not be taken as a reason for relaxation of conservation measures, especially as many threats are set to increase. For example, the growing amount of merchant shipping threatens to increase mortality rates of marine megafauna living near the surface,^5^ including various sharks and turtles,^6^ and the pace of climate warming is set to increase.^7^

Across a broad range of taxa, abundance trend indicators are a hugely useful conservation tool as they can respond to changes over short timescales and may be used to aggregate species trends from global down to subnational or even local scales.^8^ Traditionally, the conservation status of sea turtles has been assessed by the International Union for the Conservation of Nature (IUCN) (https://www.iucnredlist.org/) that typically assess changes in abundance over long periods (multiple generations), with their conservation threat status for different species being updated every few decades, e.g., in the study by Seminoff.^9^ These assessments are hugely important, underpinning conservation measures such as bans on international trade for critically endangered species. However, a shortcoming is that these IUCN assessments are only updated infrequently and so may not quickly capture emerging conservation concerns. At the same time, groups around the world publish information in the public domain on abundance of turtles at their particular study sites. These time series can provide a useful complement to the periodic IUCN assessments. For example, in 2017, a study examined 299 abundance time series for sea turtles and showed that generally upward abundance trends dominated.^4^

Here, we compile the latest time series of abundance for sea turtles around the world, i.e., time series not previously examined within a global analysis. In this way, we provide an up-to-date pulse check for general trends in sea turtle abundance using the latest information. We set out to address 3 questions: (i) has the general tendency for increases in sea turtle abundance been maintained in newly published time series? (ii) are there emerging concerns where previously increasing sea turtle numbers are now decreasing? and (iii) are there any consistent patterns of population increases or decreases, such as between species and regions?

## Results

**Figure 1:**
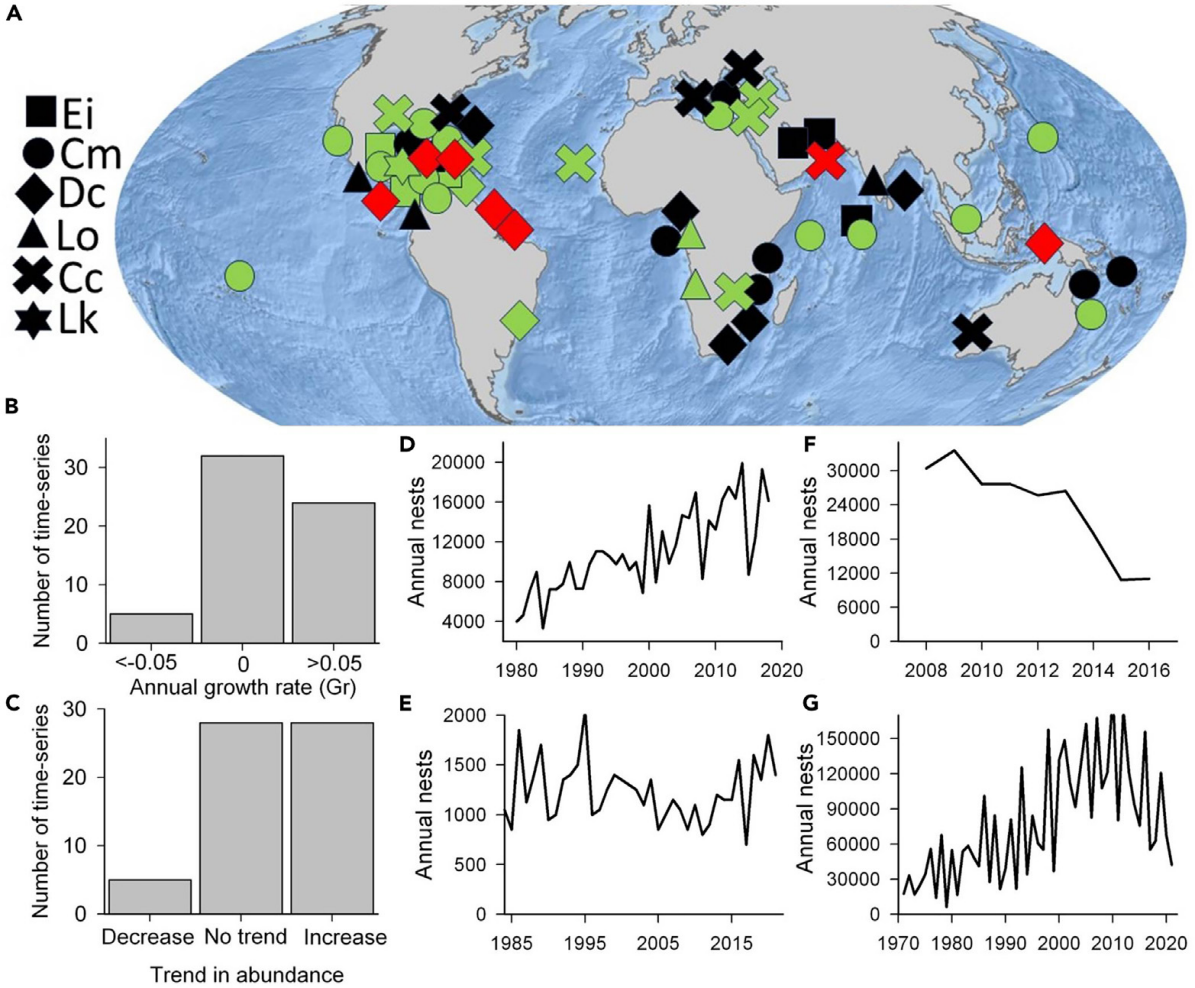
Recent trends in the abundance of turtles across the world (A) The location of recent time series of abundance with upward trends (green), downward trends (red), and no change (black), with species denoted by the symbol shape. Ei = *Eretmochelys imbricata* (hawksbill turtle), Cm = *Chelonia mydas* (green turtle) Dc = *Dermochelys coriacea* (leatherback turtle), Lo = *Lepidochelys olivacea* (olive ridley turtle), Cc = *Carretta caretta* (loggerhead turtle), *Lepidochelys kempii* (Kemp’s ridley turtle). Points are jittered to reduce overlap. For entire time series, (B) the annual growth rate (Gr, proportion of population change per year) and (C) the number of time series that show a significant decrease, no trend, or significant increase for the entire times series. Abundance time series for different species and areas. (D) The increase in nest numbers for green turtles at Aldabra (Seychelles, Indian Ocean), (E) no trend in loggerhead turtle nest numbers in Zakynthos (Greece), (F) the decrease in loggerhead turtle annual nests at Masirah Island (Oman), and (G) the reversal of an upward to a downward trend in abundance for green turtles at Tortuguero (Costa Rica).

We examined 61 time series of annual abundance (Table S1) that varied in length from 6 to 57 years (mean 20 years, SD = 12 years) and spanned >1200 years of monitoring (Figure 1A). Annual growth rates indicated that most populations were either increasing or relatively stable (Figure 1B) and this conclusion was reiterated by trend analysis that showed of the 61 time series examined, upward population trends and no change were most common (28 and 28 time series, respectively) and downward trends were rare (5 time series) (Figure 1C). Across all species, there were examples of increases in abundance but significant decreases in abundance only occurred in leatherbacks and loggerheads. For example, the proportion (and %) of stable or upward trends in abundance were: hawksbill 7 of 7 (100%), green 19 of 19 (100%), leatherback 12 of 16 (75%), olive ridley 5 of 5 (100%), loggerheads 12 of 13 (92%), and Kemp’s ridley 1 of 1 (100%). Similarly, stable or upward trends dominated across ocean basins: 18 of 19 time series in the Caribbean, 9 of 11 time series in the Pacific, 12 of 12 time series in Indian Ocean, 6 of 6 time series in the Mediterranean, 2 of 3 time series in the Arabian Sea, and 9 of 10 time series in the Atlantic.

Upward trends in abundance include green turtles at Aldabra (Seychelles, Indian Ocean) where annual nests have risen from around 4000 in 1980 to around 16,000 in 2018 (Figure 1D), loggerhead turtles in Sal (Cape Verde, north Atlantic) where annual nests have risen from around 500 in 2008 up to 35,000 in 2020, and Kemp’s ridley turtles in Mexico where annual nests have risen from around 2000 in 1996 to around 17,000 in 2022. Many time series showed relatively stable nesting numbers, such as loggerhead turtles in Zakynthos (Greece, Mediterranean) (Figure 1E) and leatherback turtles in South Africa (Indian Ocean).

**Figure 2:**
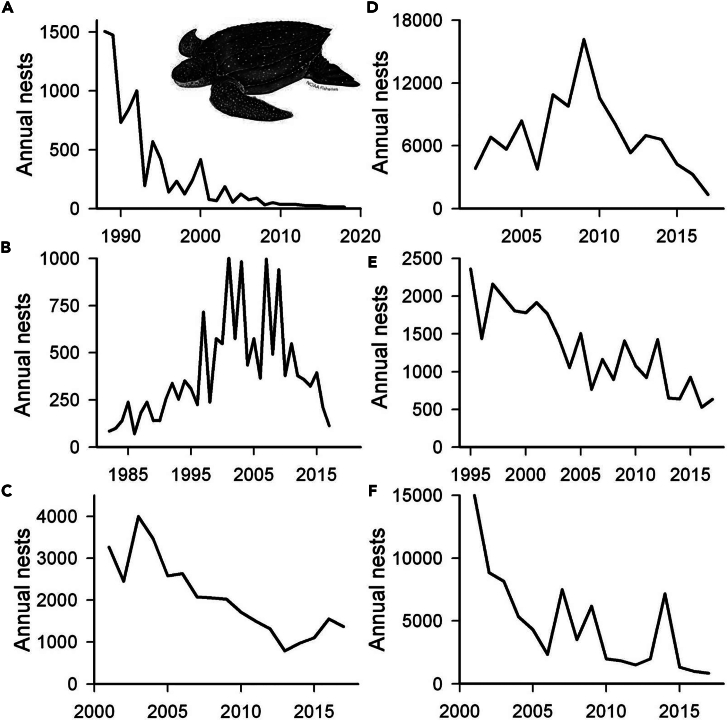
Examples of declines in leatherback turtle abundance In some areas, there have been sustained long-term declines in nesting abundance of leatherback turtles, while in other areas upward or stable trends have reversed to downward trends. (A) Las Baulas (Pacific coast of Costa Rica), long-term decline; (B) St Croix (Caribbean, US Virgin Islands), recent decline; (C) Jamursba Medi (Indonesia, western Pacific), long-term decline; (D) French Guiana (Atlantic), recent decline; (E) Tortuguero and Pacuare (Costa Rica, Caribbean), long-term decline; (F) Suriname (Atlantic), long-term decline. Image of leatherback turtle courtesy of NOAA.

A significant downward trend has occurred for loggerhead turtles at Masirah Island (Oman) where annual nests have declined from around 30,000 to 10,000 between 2008 and 2016 (Figure 1F) and for several leatherback turtle sites (Figure 2) including Las Baulas (Pacific coast of Costa Rica) where the number of nesters has declined from around 1,500 to 15 between 1988 and 2018, Suriname (Atlantic) where the annual number of nests has declined from around 10,000 to around 1000, as well as Indonesia (western Pacific) and the Caribbean coast of Costa Rica. There were three clear cases where stable or upward trends had reversed to a downward trend in the most recent 10 years: for green turtles on the Caribbean coast of Costa Rica (Tortuguero) (Figure 1G), for leatherbacks in the US Virgin Islands (St Croix, Caribbean), and for leatherbacks in French Guiana (Atlantic) (Figures 2B and 2D).

## Discussion

### Trends in abundance

Our pulse check makes important advances on a previous pulse check published in 2017.^4^ Although we demonstrate prevailing upward or stable trends in annual sea turtle nesting globally, indicating the potential for rebuilding marine megafauna populations across large spatial scales despite growing threats,^1^^,^^3^ new concerns are highlighted. First, formerly major populations can go into decline, such as loggerhead turtles nesting in Oman^10^; second, previous upward or stable trends can turn into declines such as with green turtles nesting at Tortuguero (Costa Rica)^11^ and leatherback turtles nesting in French Guiana^12^; third, the compilation of abundance time series for leatherback turtles has shown that several populations, often where nesting was formerly very abundant, are experiencing steep declines.^12^ These observations show the importance of time series of abundance for both identifying conservation successes but also triaging where declines in abundance highlight that conservation management solutions are needed.

We showed many notable increases in abundance for sea turtles across species and regions as well as many stable populations. This great news for sea turtle conservation likely reflects a range of conservation measures being implemented, including national directives (e.g., the creation of marine protected areas, fishing regulations, and conservation enforcement) to grass roots conservation driven by non-governmental organizations, such as the relocation of threatened clutches into hatcheries and nightly beach patrols to both collect census information as well as discouraging poaching. However, threats to turtles remain. For example, in the Commonwealth of the Northern Mariana Islands (northern Pacific), an annual increase in nesting numbers of green turtles of 7.4% has been reported but elegant modeling suggests that without poaching of nesting females this growth rate would be around 11.4%.^13^ Further, a primary driver of turtle population recovery in many countries may be the reduced exploitation for meat, soup, leather, and shells. Here, the impact of the Convention on International Trade in Endangered species of Wild Fauna and Flora and supportive protective national legislation should be highlighted as important vehicles that likely have a profound impact on sea turtle conservation.

Examples of increases in annual abundance include increases in annual green turtle clutches and or number of nesting females reported for Príncipe Island (São Tomé, West Africa) using monitoring data from 2007 to 2015,^14^ Mexico (southern Gulf of Mexico) using data from 1990 to 2021,^15^ Redang Island (Malaysia) from data collected between 1993 and 2019,^16^ and on Diego Garcia (Chagos Archipelago, Indian Ocean)^17^ although infrequent monitoring at this remote site may mask trends. Across a 22-year period, significant increases in nesting numbers for green turtles have been reported in the Cayman Islands, with, for example, an increase in green turtle nests on Grand Cayman from 82 to 1,005 representing a 1126% increase.^18^ Encouragingly, major nesting sites continue to be described for the first time in the scientific literature. For example, only recently have the trends in nesting activity for green turtles in New Caledonia been published.^19^ A nesting population of amazing size was described, with >100,000 tracks in some years likely making this one of the largest nesting populations in the Pacific.

Increases in nest abundance for loggerhead turtles have been reported, for example, in Mexico from monitoring data collected between 1995 and 2018,^20^ in Turkey using monitoring data between 2001 and 2020,^21^ and in Cape Verde using data from 2008 to 2020.^22^ Across core index beaches in Florida, no long-term trend in nesting numbers was reported between 1989 and 2018, with nesting numbers displaying a cyclical pattern.^23^ Similarly, at Zakynthos (Greece), one of the largest nesting areas in the Mediterranean, a stable number of nests was reported between 1984 and 2021.^24^ Increases in loggerhead turtle nest numbers have been reported in the Cayman Islands.^18^ Increases in the number of nests for olive ridley turtles have been found on the Pacific coast of NW Mexico^25^ from analysis of monitoring data collected across 2 beaches from 1989 to 2016. A trend for relatively stable or slight increase in nest numbers has been reported for olive ridley turtles on the coast of Angola, which hosts the largest nesting population of this species in the Atlantic as well as the largest non-arribada population globally.^26^ Relatively stable nest numbers for olive ridley turtles have been reported on the Pacific coast of Costa Rica, where there are more than 1 million nests in some years.^27^ In Gabon and Congo (Atlantic coast of central Africa), an increase in the number of nesting olive ridley turtles has been reported.^28^

### Drivers of increases in abundance

The drivers of upward population trajectories are likely broadly divided into (i) increases in survival of eggs and adults on and close to nesting beaches and (ii) increases in survival on distant foraging grounds.^29^ It is likely that conservation measures in both these broad areas have played a role in upward trends. For example, at Cape Verde (Atlantic Ocean), the dramatic increase in the annual number of loggerhead turtle nests has been attributed, at least partly, to conservation efforts reducing the harvesting of eggs and nesting adults on beaches.^22^ In the Gulf of Mexico where green turtles were historically heavily exploited through a targeted fishery, a >20-fold increase in their at-sea abundance between 1995 and 2018 points to hugely positive conservation outcomes from efforts to reduce mortality on these foraging grounds.^30^ These case studies show how there may be multiple routes to successful conservation involving both protection of sea turtles on their nesting beaches, including reducing the mortality of both nesting females and their eggs, as well as on their foraging grounds, such as reduced take in turtle fisheries.^30^

The extent of increases in abundance is particularly startling at some sites, with >10-fold increases in annual abundance reported at some sites in recent decades, such as the island of Sal (Cape Verde, Atlantic) where the number of nests increased from about 500 in 2008 to 35,500 in 2020.^22^ Such examples provide clear evidence that major increases in nesting numbers can be achieved within a few decades, even from formerly small nesting populations. There are other recent indications of the success of conservation measures. For example, in the Chagos Archipelago (Indian Ocean) where immature sea turtles have been protected for many decades and the area lies within a massive marine protected area, the largest density of immature foraging hawksbill turtles anywhere in the world has recently been recorded using drone surveys, likely a consequence of decades of protection at this site.^31^

### Causes for concern

While the increasing and stable trends in annual nesting abundance for many species around the world are heartening, leatherback turtles show some alarming declines. The startling decline in nesting numbers of leatherback turtles in Eastern Pacific (Playa Grande, Costa Rica) continues unabated, despite conservation efforts on the nesting beaches.^12^ Further, leatherbacks are showing declining trends in abundance at several other sites including Indonesia, French Guiana, and Suriname.^12^ Furthermore, at some areas where they were formally abundant, such as nesting beaches in Malaysia, leatherbacks have almost completely disappeared and are virtually extinct.^12^ In addition to consumption of eggs and nesting turtles, these declining trends in leatherback abundance may be linked to the broadscale movements of individuals, sometimes across many thousands of kilometers spanning ocean basins,^32^ where they are exposed to a number of threats including bycatch in oceanic longline fisheries as well as coastal pot fisheries.^33^ Innovative solutions to reduce bycatch across this range of fisheries may be key to reducing mortality rates.^34^ Encouragingly, some leatherback turtle populations are increasing such as on nesting beaches in Brazil and in Grenada (southern Caribbean).^35^ These trends are occurring despite the likely large-scale movements of individuals exposing them to a range of fisheries.^36^ If the reasons can be identified why leatherback turtle abundance is increasing or stable in some areas versus decreasing in other areas, it may help triage the most important threats in need of mitigation to reverse declines.

Declines in abundance are not limited to leatherback turtles. For instance, a recent study has revealed a long-term decline in abundance at Masirah Island, Oman, formerly thought to be the largest loggerhead turtle nesting colony in the world, linked to increasing harvesting.^10^ These declines in abundance highlight the need to continued conservation measures across the world to protect recent conservation gains against emerging threats. It is also important to remember that despite increases in abundance in recent decades, turtle numbers in some areas may still be lower than they were in former times >50 years ago. For example, this point has been noted for Kemp’s ridley turtles in Mexico.^37^ Further, for some major nesting sites, there are grave conservation concerns that suggest nest numbers may soon decline catastrophically. For example, at Raine Island (Australia), high embryo mortality at what has been described as the world largest green turtle rookery increasingly threatens population viability.^38^

### Limitations of the study

We note that a caveat of our general conclusion of stable or upward trends is that many, perhaps even most, sea turtle nesting beaches do not have recent published time series of nesting numbers. Furthermore, it may be that those nesting beaches that are monitored, and hence have abundance time series, might also be the ones that receive most protection due to the presence of turtle conservation biologists. A further caveat of the available time series is that they are not equally spread across the world. There is a relative abundance of time series in some areas, such as the Caribbean, but other areas have fewer time series, such as parts of Africa. Hence, we reiterate previous sentiments^4^ that more abundance time series will allow improved estimates of the global status of sea turtles.

In some cases, it may be difficult to monitor nesting abundance on beaches. For example, some sites are remote and rarely visited during the nesting season.^16^ So there may be different data qualities in the available time series, which may affect the ability to detect long-term trends. Furthermore, the quality of the data underlying published reports may not always be clear. In addition, inter-annual variability in nesting numbers may mask long-term trends.^39^ So both measures of data quality, as well as longer time series, will help improve future global assessments of abundance trends. Nevertheless, our key conclusion is likely robust, namely that mostly abundance time series show upward trends or no change, with the exception of leatherback turtles where often trends are downward.

The individual time series we have examined clearly do not cover all the nesting sites and data gaps remain. Further, our data are not directly comparable with IUCN assessments that tend to assess trends in abundance integrated over larger spatial scales spanning many counties, e.g., in the study by Mancinni et al.^40^ Nevertheless, our global assessment serves as a useful pulse check of the status of sea turtles, supplementing the more intermittent and broader brush IUCN assessments.

### The value of ongoing monitoring

While in both terrestrial and marine ecosystems protected areas may help reduce declines in vertebrate abundance,^41^^,^^42^ it has been widely noted that the broadscale movements of sea turtles, often across national borders, occurring both as part of developmental and breeding migrations, means that individual turtles are unlikely to be within protected areas for their entire lives or even just within their adult lives (e.g., Ref. 43). So the increases in abundance we found across species and oceans basins are even more remarkable and point to conservation measures acting successful across the broad areas occupied by sea turtles. Regular pulse checks of the status of sea turtles will be important to identify emerging threats as well as show where conservation measures are working well and need to be continued.

## STAR★Methods

### Key resources table

**Table:** 

REAGENT or RESOURCE	SOURCE	IDENTIFIER
Annual abundance number	Literature value	Available in Supplementary

**Software**		

Mintab Ver. 8.2 Ext	Minitab	https://www.minitab.com

### Resource availability

#### Lead contact

Further information and requests for resources should be directed to and will be fulfilled by the lead contact: Professor Graeme Hays (g.hays@deakin.edu.au).

#### Materials availability

This study did not generate new unique reagents.

#### Data and code availability

All data reported in this paper are available in the supplemental information.

This paper does not report original code.

Any additional information required to reanalyze the data reported in this paper is available from the lead contact upon request.

### Method details

Following the procedures detailed previously,^4^ we assembled recent time series of abundance of sea turtles at different sites. To do this we used published articles of annual abundance, using the values given within tables in those articles or digitising values if they appeared only in graphs. Only time series that included annual abundance to 2015 and beyond and that had not been included in a previously pulse check^4^ were used. All time series reported the number of nests or number of nesting females, with one exception (Gulf of Mexico) that included at-sea catch per unit effort^30^ (Table S1). Only studies with a minimum of 6-years of data were included. Where there were counts of numbers across beaches in one country, then these were added to provide a single country value. The exceptions were for Costa Rica, where beaches on the Caribbean and Pacific coasts were kept separate and widely separated sites in the USA and Australia. The longest time series from individual countries were used and data across nesting beaches only merged where individual time series covered the same years.

### Quantification and statistical analysis

We used linear regression models to detect directional upward or downward trends in each time series (p < 0.05). Since interannual variability in numbers might sometimes mask significant trends, regardless of whether there were significant upward or downward trends in abundance we also calculated the annual growth rate for each time series to provide another approach of detecting change. The annual growth rate was calculated from the mean abundance in the last 3 years of each time series (*N*_*L*_) compared to the mean abundance in the first 3 years of the same time series (*N*_*F*_) and the length of time series (*n* years) using the equation:

$$\text{Annual}\;\text{Growth}\;\text{Rate}\;\left(\text{Gr}\right)={\left(N_{L}\;/\;N_{F}\right)}^{\left(1/n-3\right)}\;–\;1$$

## Supplemental information

Table S1. Abundance time series for sea turtles around the world related to Figure 1 Time-series are an index of the number of nests or number of nesting females, with one exception (Gulf of Mexico) that included at-sea catch per unit effort on foraging grounds.
